# Steady-State Serum IgG Trough Levels Are Adequate for Pharmacokinetic Assessment in Patients with Immunodeficiencies Receiving Subcutaneous Immune Globulin

**DOI:** 10.1007/s10875-021-00990-z

**Published:** 2021-05-26

**Authors:** Zhaoyang Li, Barbara McCoy, Werner Engl, Leman Yel

**Affiliations:** 1 Shire Human Genetic Therapies, Inc., a Takeda company, 650 East Kendall Street, Cambridge, MA 02142 USA; 2 Baxalta Innovations GmbH, a Takeda company, Vienna, Austria; 3 Baxalta US Inc., a Takeda company, Cambridge, MA USA

**Keywords:** Pharmacokinetics, primary immunodeficiency diseases, subcutaneous immunoglobulin, intravenous immunoglobulin, Cuvitru

## Abstract

Patients with primary immunodeficiency diseases often require lifelong immunoglobulin (IG) therapy. Most clinical trials investigating IG therapies characterize serum immunoglobulin G (IgG) pharmacokinetic (PK) profiles by serially assessing serum IgG levels. This retrospective analysis evaluated whether steady-state serum IgG trough level measurement alone is adequate for PK assessment. Based on individual patient serum IgG trough levels from two pivotal trials (phase 2/3 European [NCT01412385] and North American [NCT01218438]) of weekly 20% subcutaneous IG (SCIG; Cuvitru, Ig20Gly), trough level-predicted IgG AUC (AUC_τ,tp_) were calculated and compared with the reported AUC calculated from serum IgG concentration-time profiles (AUC_τ_). In both studies, mean AUC_τ,tp_ values for Ig20Gly were essentially equivalent to AUC_τ_ with point estimates of geometric mean ratio (GMR) of AUC_τ,tp_/AUC_τ_ near 1.0 and 90% CIs within 0.80–1.25. In contrast, for IVIG, 10%, mean AUC_τ,tp_ values were lower than AUC_τ_ by >20%, (GMR [90% CI]: 0.74 [0.70–0.78] and 0.77 [0.73–0.81] for the two studies, respectively). Mean AUC_τ,tp_ values calculated for 4 other SCIG products (based on mean IgG trough levels reported in the literature/labels) were also essentially equivalent to the reported AUC_τ_ (differences <10% for all except HyQvia, a facilitated SCIG product), while differences for IVIG products were >20%. In conclusion, steady-state serum IgG levels following weekly SCIG remain stable, allowing for reliable prediction of AUC over the dosing interval using trough IgG levels. These findings indicate that measuring steady-state serum IgG trough levels alone may be adequate for PK assessment of weekly SCIG.

**Supplementary Information:**

The online version contains supplementary material available at 10.1007/s10875-021-00990-z.

## Introduction

Primary immunodeficiency diseases (PID) are a heterogeneous group of >400 disorders that are often characterized by absent or deficient antibody production, which can lead to frequent and severe infections [1, 2]. Patients with PID often require lifelong immunoglobulin (IG) replacement therapy, which reduces the frequency and severity of infections [3, 4]. IG replacement therapy is administered intravenously (IVIG; every 3–4 weeks) or subcutaneously (SCIG; usually daily to every 2 weeks) [5]. Unlike IVIG, SCIG products can be self-administered at home, are associated with a lower risk of systemic adverse events, and do not require venous access [3–5]. The benefits of SCIG use in patients with immunodeficiencies have led to the development of several SCIG products with various concentrations of immunoglobulin G (IgG) [1].

The pharmacokinetic (PK) properties of exogenous IG vary by route of administration. While the infusion of IVIG treatment directly into the intravascular space results in an early, high peak of IgG serum concentration (*C*_max_) followed by distribution and elimination phases, the slower absorption of SCIG from the SC infusion site results in a more gradual and stable increase in IgG serum concentration for several days, with a lower *C*_max_ than the peak achieved with IVIG infusions [5–7]. The increased dosing frequency of SCIG compared with IVIG may contribute to the more stable IgG levels, and less fluctuation between peak and trough of steady-state IgG levels throughout the dosing cycle and the occurrence of higher trough IG levels compared with IVIG [6, 8].

Serum IgG trough level monitoring is an important consideration when evaluating IG therapies. While patients show considerable interindividual variability in trough IgG levels, higher serum IgG trough levels (into the normal range for IgG) have been associated with a decreased risk of infections and improved clinical outcomes [4, 9]. PK assessments have also been deemed essential by health authorities to support the pharmacological activity and efficacy in the registration of IG products. The US Food and Drug Administration recommends that investigational IG products be evaluated for PK parameters, including the area under the curve (AUC) of serum IgG, for comparisons with effects of previous IVIG treatment [7, 10]. Dose adjustment of SCIG products may be necessary to achieve an AUC of serum IgG that is equivalent with IVIG products due to the decreased bioavailability of IG-administered SC versus IV [7]. In addition, for new IG products, the European Medicines Agency requires assessment of serum trough levels of IgG as well as other PK parameters in comparison with those achieved with the former IVIG or SCIG product [11].

Clinical investigation studies often use serial blood sampling as a means for understanding PK properties in accordance with regulatory guidance. These studies commonly characterize serum IgG PK profiles through the serial evaluation of serum IgG levels during one or more dosing intervals [12–15]. While serial sampling generally facilitates the provision of valuable PK data, this practice has some drawbacks, including added logistical challenges for the conduct of clinical trials, as well as the burden of multiple blood draws for patients, particularly for pediatric patients, for whom venipuncture can be a highly distressing experience [16]. Blood draws are one of the most common reasons that pediatric patients decline to participate in clinical trials, and the need for additional blood collection outside routine care is a concern expressed by pediatric patients as well as their caregivers [17]. For these patients and others, characterizing PK profiles with fewer blood collection time points, if demonstrated feasible and valid, can be beneficial for eliciting participation from patients in clinical trials and for trial investigators.

Serum IgG generally exhibits prolonged SC absorption, with the time to reach *C*_max_ reported in the range of days and long elimination half-life reported in weeks [18]. With weekly dosing for SCIG, peak serum IgG levels at steady state are hardly discernible, and IgG levels remain rather stable from pre-infusion throughout the treatment interval [12, 14]. Therefore, it is reasonable to believe that the IgG trough level is representative of not only the exposure level at a single time point, but also the total exposure over the dosing interval (AUC_τ_).

The objective of this retrospective analysis is to evaluate whether serum IgG trough level measurement alone can provide reliable AUC assessment over a dosing interval at steady state compared with that calculated from serial sampling.

## Methods

### PK Data Source for Ig20Gly

Individual patient PK data were obtained from 2 prospective, open-label, noncontrolled multicenter phase 2/3 licensing studies evaluating weekly Ig20Gly (Cuvitru) in patients with PID conducted in Europe (NCT01412385) [12] and North America (NCT01218438) [14]. Inclusion criteria, exclusion criteria, and study designs have previously been described [12, 14]. Briefly, patients aged ≥2 years with a documented diagnosis of PID requiring IG replacement therapy, for ≥3 months before the first study treatment, and serum IgG trough levels >5 g/L at screening were included.

Patients in the European study received IVIG, 10% for 13 weeks or SCIG, 16% for 12 weeks in period 1, and then received weekly SCIG, 20% at a dose of the period 1 dose adjusted to weekly equivalent for 52 weeks [12]. In the North American study, patients received IVIG, 10% for 13 weeks in period 1, and then received weekly SCIG, 20% at 145% of the IVIG, 10% dose adjusted to weekly equivalent for 12–28 weeks during period 2 and period 3. This was followed by an individualized dose of SCIG, 20% for 40 weeks in period 4 [14]. In both trials, IgG trough levels were assessed in all patients at defined time points throughout the course of each study period, with some additional PK serial sampling collections for patients aged ≥12 years in both of the studies [14]. Abbreviated serial PK sampling was performed for patients aged <12 years in the North American study (Supplemental Figure S1).

### PK Data Source for Other IG Products

Mean IgG trough levels were obtained from the published licensing studies for 6 other IG products (SCIG products: Kiovig/GammaGard Liquid [Baxalta US Inc., a member of the Takeda group of companies], HyQvia [Baxalta US Inc., a member of the Takeda group of companies], Gamunex [Grifols Therapeutics Inc.], and Hizentra [Grifols Therapeutics Inc.]; IVIG products: Kiovig/GammaGard and Gamunex). A description of the IG products evaluated is provided in Table 1.

**Table 1:** Summary of SCIG and IVIG products evaluated

Product	Manufacturer	Dosage form and strength	Approved method of administration and dosage in PID	IG content
Cuvitru^a^ [19]	Baxalta US Inc., a Takeda company, Lexington, MA, USA	Immune Globulin Subcutaneous (Human), 20% Solution (200 mg/mL)	SC: 1.30 × current IVIG^b^ dose (g)/IVIG^b^ dose interval (weeks); at regular intervals from daily to every 2 weeks (individualized per patient). If switching from another SCIG, administer at same dose as previous treatment	≥98% IgG with ~80 μg/mL IgA
Kiovig/GammaGard Liquid [20]	Baxalta US Inc., a Takeda company, Lexington, MA, USA	Immune Globulin Infusion (Human), 10% Solution (100 mg/mL)	IV: 300–600 mg/kg every 3–4 weeks SC: 1.37 × current IV dose (g)/IV dose interval (weeks); weekly	≥98% IgG with trace amounts of IgA (average concentration of 37 μg/mL)
HyQvia/HYQVIA [21]	Baxalta US Inc., a Takeda company, Lexington, MA, USA	Immune Globulin Infusion 10% (Human), Solution (100 mg/mL) and Recombinant Human Hyaluronidase (160 U/mL)	SC: 300–600 mg/kg every 3–4 weeks for patients naïve to or switching from another SCIG. If switching from IVIG, administer at the same dose and frequency as previous treatment	≥98% IgG with trace amounts of IgA (average concentration of 37 μg/mL)
Gamunex [22]	Grifols Therapeutics Inc., Research Triangle Park, NC, USA	Immune Globulin Injection (Human), 10% Solution	IV: 300–600 mg/kg every 3–4 weeks SC: 1.37 × current IV dose (g)/IV dose interval (weeks); weekly	≥98% IgG with trace levels of fragments, IgA (average concentration of 46 μg/mL), and IgM
Hizentra [23]	CSL Behring AG, Bern, Switzerland	Immune Globulin (Human), 20% Liquid (200 mg/mL)	SC: 1.37 × current IV dose (g)/IV dose interval (weeks); at regular intervals from daily to every 2 weeks (individualized per patient)	≥98% IgG with ≤50 μg/mL IgA

^a^Also known as Ig20Gly

^b^Or facilitated SCIG

*IG*, immunoglobulin; *Ig20Gly*, Immune Globulin Subcutaneous (Human), 20% Solution; *IgA*, immunoglobulin A; *IgG*, immunoglobulin G; *IgM*, immunoglobulin M; *IV*, intravenous; *IVIG*, intravenous immunoglobulin; *PID*, primary immunodeficiency diseases; *SC*, subcutaneous; *SCIG*, subcutaneous immunoglobulin

For IVIG and SCIG administration of Kiovig/GammaGard, PK data were obtained from a multicenter, prospective, open-label North American study of patients with PID [20, 24]. Patients received IV infusions at 3- or 4-week intervals based on their pre-study IV dosing regimen during the first study period, and then were switched to receive weekly SC infusions at varying doses (130% of IV of weekly equivalent for period 2, 137% of IV of weekly equivalent for period 3, and an individually adjusted dose for period 4) for the remainder of the study period.

For the facilitated SCIG, HyQvia/HYQVIA (Immune Globulin Infusion, 10% administered with recombinant human hyaluronidase), PK data were obtained from a prospective, open-label, noncontrolled, multicenter US trial in patients with PID who had received IVIG treatment for ≥3 months, followed by HyQvia/HYQVIA (at 108% of the weekly equivalent IV dose) administered every 3–4 weeks for approximately 14–18 months [21, 25].

For IVIG and SCIG administrations of Gamunex, PK data were obtained from an open-label, crossover North American trial in patients with PID who had previously received or were currently receiving IG replacement therapy. Patients received Gamunex 200–600 mg/kg IV every 3–4 weeks for at least 3 months, then were switched to weekly SC infusions at a dose adjustment coefficient (DAC) of 1.37 for up to 24 weeks [22, 26].

Data for Hizentra were obtained from 2 studies conducted in Europe and the USA. In the European PK substudy, patients with PID who had previously received IVIG treatment (Privigen®, Immune Globulin Intravenous [Human], 10% Liquid) were switched to weekly subcutaneous (SC) treatment with Hizentra [23, 27]. After a 3-month wash-in/wash-out period, doses were individually adjusted to achieve a systemic serum IgG exposure that was not inferior to that of the previous weekly equivalent IVIG dose. In a US multicenter, prospective, open-label study, patients with PID who had received regular intravenous (IV) treatment with Privigen for ≥3 months prior to enrollment and had achieved serum trough concentration values ≥5 g/L were switched at study entry to weekly SC treatment with Hizentra at an initial dose calculated using a DAC of 1.3 [28].

### Calculation of Trough-Predicted AUC

Trough-predicted AUC (AUC_τ,tp_) was calculated based on serum IgG trough levels using the following formula:

$$ {\mathrm{AUC}}_{\uptau, \mathrm{tp}}={C}_{\mathrm{trough},\mathrm{ss}}\times \tau $$

where *C*_trough,ss_ is the steady-state trough concentration and *τ* is the dosing interval.

AUC_τ,tp_ was compared with the reported AUC_τ_ that was derived from PK profiles, which were estimated based on serum IgG levels at various time points from serial sampling. For Ig20Gly, data for individual patients are available from the two licensing studies. Summary statistics of the AUC values, as well as the geometric mean ratio (GMR) of AUC_τ,tp_/AUC_τ_ (point estimates and 90% CIs obtained from back-transforming the log-transformed values), were calculated. In addition, Bland-Altman plots of log-transformed AUC_τ,tp_ and AUC_τ_ values were generated to assess agreement of the methods at individual levels rather than as a summary over the full sample [29].

For other SCIG or IVIG products, the calculation of AUC_τ,tp_ was based on the published mean serum trough data, and the % difference between AUC_τ,tp_ and AUC_τ_ was determined using the following formula:

$$ \%\kern.3em \mathrm{difference}=\left[\left({\mathrm{AUC}}_{\uptau, \mathrm{tp}}-{\mathrm{AUC}}_{\uptau}\right)/{\mathrm{AUC}}_{\uptau}\right]\times 100 $$

For completeness, for Ig20Gly, the mean trough data were also used to calculate AUC_τ,tp_ and the % difference between AUC_τ,tp_ and AUC_τ_.

For all studies included in this retrospective analysis, patients or their guardians provided written informed consent according to local consent procedures in accordance with the ethical standards of their respective institutional research committees and with the 1964 Helsinki declaration and its later amendments [12, 14, 24–28].

## Results

**Fig. 1:**
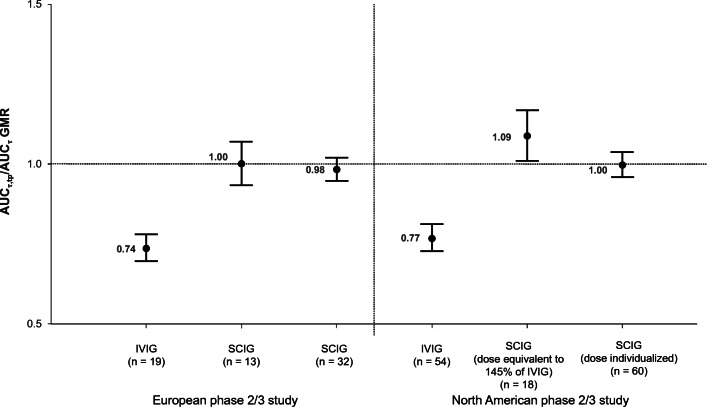
Trough-predicted AUC_τ_,_tp_ versus reported AUC_τ_ (90% CIs) for IVIG and SCIG in two phase 2/3 licensing studies of Ig20Gly (Cuvitru), calculated using individual patient data. Error bars represent 90% CIs. Horizontal reference line = GMR of 1.0. AUC_τ_, area under the curve calculated from serum IgG concentration-time profiles over a dosing interval; AUC_τ,tp_, trough level-predicted area under the curve over a dosing interval; CI, confidence interval; GMR, geometric mean ratio; Ig20Gly, Immune Globulin Subcutaneous (Human) 20% Solution; IVIG, intravenous immunoglobulin; SCIG, subcutaneous immunoglobulin; τ, dosing interval (3–4 weeks for IVIG [Kiovig/GammaGard and Gamunex] and facilitated SCIG [HyQvia/HYQVIA], and 1 week for SCIG [Cuvitru, Kiovig/GammaGard, Gamunex, and Hizentra])

**Fig. 2:**
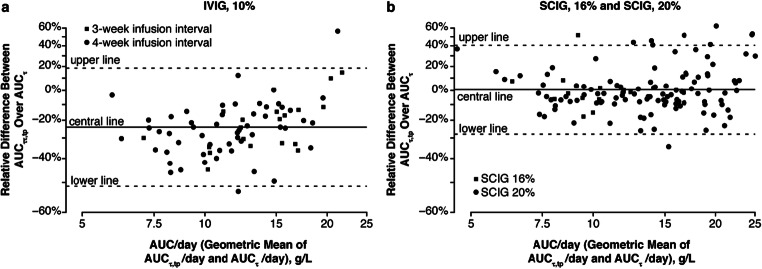
Agreement between trough-predicted AUC_τ_,_tp_ versus reported AUC_τ_ for IVIG (**a**) and SCIG (**b**) in two phase 2/3 licensing studies of Ig20Gly. To facilitate the interpretation, back-transformed values are shown. The central (upper/lower) line represents the mean (± 1.96 standard deviations) of the log-transformed difference of predicted vs reported values to account for the approximately Gaussian distribution of the AUCs. AUC_τ_, area under the curve calculated from serum IgG concentration-time profiles over a dosing interval. AUC_τ,tp_, trough level-predicted area under the curve over a dosing interval; Ig20Gly, Immune Globulin Subcutaneous (Human) 20% Solution; IVIG, intravenous immunoglobulin; SCIG, subcutaneous immunoglobulin; τ, dosing interval (3–4 weeks for IVIG, 10%, and 1 week for SCIG, 16% and SCIG, 20%)

In the two Ig20Gly licensing studies, mean values of AUC_τ,tp_ for SCIG, 16% and SCIG, 20% were essentially equivalent to the reported AUC_τ_, with point estimates of GMR of AUC_τ,tp_ versus AUC_τ_ between 0.98 and 1.09, and all 90% CIs within the commonly used equivalence limit of 0.80–1.25 (Fig. 1, Supplemental Table S1). By comparison, for IVIG, mean values of AUC_τ,tp_ in these 2 studies were consistently lower than the reported AUC_τ_ by greater than 20%; the point estimate of GMR (90% CI) of AUC_τ,tp_ versus AUC_τ_ was 0.74 (0.70–0.78) and 0.77 (0.73–0.81) for the European study and North American study, respectively (Fig. 1, Supplemental Table S1). Bland-Altman plots for IVIG, 10% (Fig. 2a) and SCIG treatment (SCIG, 16% and SCIG, 20%) (Fig. 2b) administration show the individual agreement for AUC_τ,tp_ and AUC_τ_ with back-transformed values to account for the approximately Gaussian distribution of the AUCs. Agreement was lower for IVIG, 10% than for SCIG treatment.

AUC_τ,tp_ were also calculated for other SCIG and IVIG products, based on mean serum IgG trough levels in the published licensing studies (Table 2). Differences between the calculated AUC_τ,tp_ and reported AUC_τ_ were >20% for both IVIG therapies, while for the SCIG therapies, AUC_τ,tp_ and AUC_τ_ were all within ± 10% of each other, except for HyQvia. The mean *C*_max_ was more than double the *C*_trough_ for all IVIG products, while differences between the means of *C*_max_ and *C*_trough_ for SCIG products were in the range of 1.9 to 23.7% (mostly within 15%), except for HyQvia (33.0%). For the 2 products administered as both SCIG and IVIG therapy (Kiovig/GammaGard and Gamunex), the IV products resulted in lower mean values of AUC_τ,tp_ and a greater difference between AUC_τ,tp_ and AUC_τ_ compared with the corresponding SC products, and greater mean *C*_max_ values and lower mean *C*_trough_ values than the SC products.

**Table 2:** Summary of PK parameters for SCIG and IVIG products (based on mean values from the published literature/labels)

	SCIG products							IVIG products	
Product and study	Cuvitru (European study) [12]	Cuvitru (US study) [14]	Kiovig/GammaGard [20]	HyQvia/HYQVIA [21]	Gamunex [22]	Hizentra (European study) [27]	Hizentra (US study) [28]	Kiovig/GammaGard [20]	Gamunex [26]
*N*	31–46	60	32–57^a^	60	26–36^a^	23	18	32–57^a^	26–36^a^
Generic name	Immune Globulin Subcutaneous (Human), 20% Solution (200 mg/mL)		Immune Globulin Infusion (Human), 10%	Immune Globulin Infusion (Human), 10% Liquid, with recombinant human hyaluronidase	Immune Globulin Infusion (Human), 10%	Immune Globulin Subcutaneous (Human), 20%		Immune Globulin Injection (Human), 10%	Immune Globulin Injection (Human), 10%
*C*_max_, mean (SD), mg/mL	9.82 (NA) ^d^	19.31 (NA) ^d^	13.93 (2.89)	16.07 (3.82)	12.2 (2.4)	8.26 (1.26)	16.16 (4.93)	22.40 (5.36)	21.1 (3.9)
*C*_trough_, mean (SD), mg/mL	8.73 (NA) ^d^	14.74 (NA) ^d^	12.02 (2.82)	10.77 (2.75)	11.4 (2.3)	8.10 (1.34)	13.70 (4.39)	10.50 (2.60)	9.6 (2.1)
Difference between *C*_max_ and *C*_trough_, %^b^	11.1	23.7	13.7	33.0	6.6	1.9	15.2	53.1	54.5
AUC_τ_, mean (SD), h*mg/mL	1506 (NA) ^d^	2765 (NA) ^d^	2202 (463)	2194 (504)	1900 (380)	1289 (220)	2534 (757)	2390 (546)	2095 (419)
AUC_τ,tp_, mean, h*mg/mL	1467	2476	2019	1809	1915	1361	2302	1764	1613
Difference between AUC_τ,tp_ and AUC_τ_, %^c^	−2.6	−10.4	−8.3	−17.5	0.8	5.6	−9.2	−26.2	−23.0

^a^Pharmacokinetic parameters were obtained from >1 study

^b^Calculated as (*C*_max_ − *C*_trough_)/*C*_max_ * 100

^c^Calculated as (AUC_τ,tp_ − AUC_τ_)/AUC_τ_ * 100

^d^Reported as geometric mean

*AUC*_*τ*_, area under the curve calculated from serum IgG concentration-time profiles; *AUC*_*τ,tp*_, trough level-predicted area under the curve; *C*_*max*_, maximum concentration; *C*_*trough*_, trough concentration; *GMR*, geometric mean ratio; *NA*, not applicable; *IVIG*, intravenous immunoglobulin; *SCIG*, subcutaneous immunoglobulin; *τ*, dosing interval (3–4 weeks for IVIG [Kiovig/GammaGard and Gamunex] and facilitated SCIG [HyQvia/HYQVIA], 1 week for SCIG [Cuvitru, Kiovig/GammaGard, Gamunex, and Hizentra]); *SD*, standard deviation; *US*, United States

## Discussion

This retrospective analysis sought to determine whether measurement of serum IgG trough levels alone is sufficient for steady-state PK assessment of SCIG therapies in patients who have PID. Given the PK characteristics of serum IgG after SC administration, i.e., long elimination half-life (in weeks) and prolonged SC absorption (in days), it is expected that the serum levels of IgG over a 1-week dosing interval at steady state would remain stable, with essentially no discernible peak in serum IgG levels. This is demonstrated by the data for Ig20Gly [12, 14] and other conventional SCIG products where the means of *C*_max_ and *C*_trough_ for SCIG products were in the range of 1.9 to 23.7% (mostly within 15%) of each other. By comparison, the mean *C*_max_ was more than double the *C*_trough_ for all IVIG products. This minimal fluctuation between peak and trough concentrations after SC administration of a SCIG therapy at steady state has formed the foundation of this proposed trough-based PK assessment.

AUC_τ,tp_ calculated based on steady-state trough levels for Ig20Gly administered weekly was found to be equivalent to the AUC_τ_ reported in pivotal studies using a serial sampling method [12, 14]. The agreement between AUC_τ,tp_ and AUC_τ_ for Ig20Gly treatment was found to be as expected given a generally reported range of 15–20% for the coefficient of variation of IgG assays. Similarly, for other SCIG products, when AUC_τ,tp_ was derived from mean serum trough IgG levels, it was within ± 10% of the reported AUC_τ_ for each individual product. In contrast, the AUC_τ,tp_ for IVIG and facilitated SCIG products was considerably lower than the reported AUC_τ_, and the agreement between AUC_τ,tp_ and AUC_τ_ was much lower for IVIG, 10%. These findings suggest that for SCIG therapies, steady-state serum IgG trough levels are representative of not only the exposure level at a single time point but also the total exposure over the dosing interval (AUC_τ_); therefore, the measurement of serum trough level alone at steady state may be adequate for PK assessment of weekly conventional SCIG treatment in patients with PID.

HyQvia/HYQVIA is a facilitated SCIG product, which is co-administered with recombinant human hyaluronidase, an absorption enhancer [21]. It is likely that the increased absorption rate of serum IgG associated with this treatment together with the dosing schedule of 3–4 weeks would result in higher *C*_max_ and a consequently larger difference between *C*_max_ and *C*_trough_, and therefore contributed to our findings for this product that trough-predicted AUC_τ,tp_ is not equivalent to the reported AUC_τ_. Similarly, the larger difference between AUC_τ,tp_ and the reported AUC_τ_ observed for IVIG products is likely the result of their increased bioavailability relative to SCIG products, increasing *C*_max_, and the longer dosing interval, which allows for more IgG to be metabolized before the next dose is administered, lowering *C*_min_ [5–8]. Given the larger variation in serum IgG levels from peak to trough during the 3–4-week dosing interval for HyQvia/HYQVIA and across IVIG products, IgG trough levels alone are not sufficient for PK assessment at steady state in patients receiving IVIG or HyQvia/HYQVIA.

Despite the fact that other PK parameters, such as volume of distribution, elimination half-life, and clearance, are occasionally reported for SCIG, these parameters are not truly accurately estimated from a steady-state PK profile over a 1-week dosing interval due to the prolonged absorption and long elimination half-life. In the authors’ opinion, the systemic exposure parameters, including *C*_trough_ and AUC_τ_, are the most relevant PK assessment at steady state for SCIG products, and our proposed serum trough alone approach will cover both the single-point exposure and the total exposure. Therefore, the value of characterizing PK profiles of serum IgG over the 1-week dosing interval by serial sampling is very limited.

One limitation of this analysis is that because this was a retrospective analysis, direct access to individual patient data was not available for all products; in these cases, the analysis was conducted only on the mean values reported in the published literature, and a Bland-Altman plot could not be provided. In addition, conclusions about the value of measuring serum IgG trough levels alone for PK assessment of SCIG products at steady state are limited in this manuscript to SCIG treatment received on a weekly basis, regardless of the dose level (100–145% of the equivalent prior IVIG dose in the Ig20Gly studies).

In conclusion, steady-state serum IgG levels remain stable following weekly SCIG treatment in patients with PID, enabling reliable prediction of total exposure (AUC_τ_) using serum IgG trough levels alone. Our findings appear to be generalizable across all conventional SCIG products. These results indicate that measuring steady-state serum IgG trough levels alone for PK assessment of weekly SCIG treatment is a reasonable and beneficial alternative to serial PK sampling during clinical development and beyond. For patients and investigators, the use of steady-state IgG trough levels for PK assessment offers multiple benefits, including more efficient clinical trial conduct through decreases in study costs and logistical complexity and, more importantly, a reduction in the patient burden of frequent blood sampling.

## Supplementary Information

ESM 1 (DOCX 37 kb)

## Data Availability

The datasets, including the redacted study protocol, redacted statistical analysis plan, and individual participants data supporting the results reported in this article, will be made available within 3 months from initial request, to researchers who provide a methodologically sound proposal. The data will be provided after its de-identification, in compliance with applicable privacy laws, data protection, and requirements for consent and anonymization.
