# Supplementary material for: Steady-State Serum IgG Trough Levels Are Adequate for Pharmacokinetic Assessment in Patients with Immunodeficiencies Receiving Subcutaneous Immune Globulin
Source: J Clin Immunol. 2021 May 26;41(6):1331–8. doi: 10.1007/s10875-021-00990-z (PMC8310849; doi:10.1007/s10875-021-00990-z)
Supplement: Supplementary file 1 — (DOCX 37 kb) [file 10875_2021_990_MOESM1_ESM.docx]

**Steady-State Serum IgG Trough Levels Are Adequate for Pharmacokinetic Assessment in Patients with Immunodeficiencies Receiving Subcutaneous Immune Globulin**

Zhaoyang Li, PhD^1^; Barbara McCoy, PhD^2^; Werner Engl, PhD^2^; Leman Yel, MD^3,4^

*^1^Shire US Inc., a Takeda company, Cambridge, MA, USA; ^2^**Baxalta Innovations GmbH, a Takeda company, Vienna, Austria; ^3^Baxalta US Inc., a Takeda company, Cambridge, MA, USA; ^4^University of California, Irvine, CA, USA*

**Corresponding Author:** Zhaoyang Li, PhD

Email: zhaoyang.li@takeda.com

Phone: (617) 588-8250

Fax: (617) 588-9030

Address:

650 East Kendall Street

Cambridge, MA 02142, USA

**Supplemental Materials**

**Supplemental Table S1.** GMR (point estimates and 90% CIs) for trough-predicted AUC_τ,tp_ versus reported AUC_τ_ in two phase 2/3 licensing studies of Ig20Gly (Cuvitru) (calculated using individual patient data)

| **Study** | **Treatment** | **N** | **AUC_τ_, hr*mg/mL** | | | **AUC_τ,tp_, hr*mg/mL** | | | **AUC_τ,tp_/AUC_τ_, GMR** | |
| --- | --- | --- | --- | --- | --- | --- | --- | --- | --- | --- |
|  |  |  | **Mean** | **SD** | **% CV** | **Mean** | **SD** | **% CV** | **Point Estimate** | **90% CI** |
| European phase 2/3 study | IVIG, 10% | 19 | 276.21 | 59.28 | 21.46 | 203.21 | 44.49 | 21.90 | 0.736 | 0.696–0.779 |
|  | SCIG, 16% | 13 | 77.42 | 28.38 | 36.65 | 76.70 | 24.55 | 32.01 | 1.001 | 0.935–1.071 |
|  | SCIG, 20% | 32 | 63.51 | 18.65 | 29.37 | 61.75 | 15.97 | 25.87 | 0.983 | 0.946–1.021 |
| North American phase 2/3 study | IVIG, 10% | 54 | 401.68 | 88.64 | 22.07 | 316.63 | 106.60 | 33.67 | 0.767 | 0.726–0.811 |
|  | SCIG, 20% (dose adjusted to 145% of IVIG 10% weekly equivalent) | 18 | 110.61 | 23.28 | 21.05 | 122.48 | 36.30 | 29.64 | 1.088 | 1.011–1.170 |
|  | SCIG 20% (dose individualized) | 60 | 117.64 | 24.26 | 20.63 | 118.42 | 30.62 | 25.86 | 0.997 | 0.958–1.037 |

AUC_τ_, area under the curve calculated from serum IgG concentration-time profiles; AUC_τ,tp_, trough level-predicted area under the curve; CI, confidence interval; CV, coefficient of variance; GMR, geometric mean ratio; Ig20Gly, Immune Globulin Subcutaneous (Human) 20% Solution; IVIG, intravenous immunoglobulin; SCIG, subcutaneous immunoglobulin; τ, dosing interval (3–4 weeks for IVIG [Kiovig/GammaGard and Gamunex] and facilitated SCIG [HyQvia/HYQVIA], 1 week for SCIG [Cuvitru, Kiovig/GammaGard, Gamunex, and Hizentra]); SD, standard deviation

**Supplemental Figure S1.** Design of Ig20Gly (Cuvitru) Phase 2/3 Licensing Studies

^a^Treatment in Period 3 started as soon as the “adjusted dose,” which was 145% of IVIG 10%, was identified. Consequently, patients in Period 1 who had enrolled after the adjusted dose information had become available entered directly into Period 3

Ig20Gly, Immune Globulin Subcutaneous [Human] 20% Solution; IVIG, intravenous immunoglobulin; PK, pharmacokinetic; SCIG, subcutaneous immunoglobulin; y, years
